# Clustering lung function and symptom profiles for asthma risk stratification

**DOI:** 10.1038/s41598-025-32977-w

**Published:** 2025-12-24

**Authors:** Alex Cucco, Angela Simpson, Clare Murray, Graham C. Roberts, John W. Holloway, S. Hasan Arshad, Adnan Custovic, Sara Fontanella

**Affiliations:** 1 National Heart and Lung Institute, Imperial College London, Hammersmith Campus, Du Cane Road, London, W12 0NN UK; 2 Department of Socio-Economic, Managerial, and Statistical Studies, University “G. d’Annunzio” of Chieti-Pescara, Pescara, Italy; 3 Division of Infection, Immunity and Respiratory Medicine, School of Biological Sciences, Faculty of Biology, Medicine and Health, University of Manchester, Manchester Academic Health Science Centre, Manchester, UK; 4 Clinical and Experimental Sciences, Faculty of Medicine, University of Southampton, Southampton, UK; 5 NIHR Southampton Biomedical Research Centre, University Hospital Southampton, Southampton, UK; 6 David Hide Asthma and Allergy Centre, St Mary’s Hospital, Isle of Wight, Newport, UK; 7 Human Development & Health, Faculty of Medicine, University of Southampton, Southampton, SO17 1BJ UK

**Keywords:** Asthma, Childhood, Bayesian profile regression, Birth cohorts, Disease subtyping, Diseases, Immunology, Medical research

## Abstract

Asthma is a heterogeneous condition often studied through wheeze alone, yet the interplay between lung function and reported symptoms remains underexplored. To capture this heterogeneity, we applied Bayesian Profile Regression to data from school-age children in two prospective birth cohorts, integrating airway hyperresponsiveness, lung function, bronchodilator reversibility, allergic sensitisation, reported symptoms, and physician diagnosis. In the Manchester Allergy and Asthma Study (discovery cohort), five reproducible clusters were identified: HA-LLF (high asthma-low lung function), HA-NLF (high asthma normal lung function), LA-RLF (low asthma-reduced lung function), LA-NLF (low asthma normal lung function), and MA-NLF (moderate asthma normal lung function). The HA-LLF and HA-NLF clusters had very high asthma prevalence (80–100%), but differed markedly in lung function, airway responsiveness, bronchodilator reversibility, sensitisation, and symptom burden. The LA-RLF and LA-NLF clusters with low asthma prevalence (< 5%) displayed contrasting lung function profiles, while MA-NLF (~ 50% asthma prevalence) was largely defined by prominent symptoms such as chest tightness and shortness of breath. These subtypes were replicated in an independent cohort, Isle of Wight. Our findings demonstrate that integrating physiological, immunological, and symptom-based measures yields clinically meaningful asthma subtypes beyond wheeze-based definitions and may support more precise disease classification.

**Supplementary Information:**

The online version contains supplementary material available at 10.1038/s41598-025-32977-w.

## Introduction

Understanding of the diverse manifestations of asthma to accurately identify subgroups of patient with distinct mechanisms is essential to advance personalised treatment^1–4^. Unbiased, hypothesis-generating methods can improve the process of distinguishing these subgroups^5–13^. A progress has been made in exploring the heterogeneity of childhood asthma using data-driven approaches, especially latent class analysis of longitudinal information on presence or absence of wheezing^14,15^. Different longitudinal patterns of symptom may point to varied underlying causes and biological processes^16–18^. However, although wheeze phenotypes in different studies are generally assigned the similar/same name (such as transient, persistent and late-onset), they often differ in symptom patterns, phenotype prevalence, and associated risk factors^19,20^. This variability within phenotypes, along with the potential for inaccurate classification^7,21,22^, may result in inconsistent links between phenotypes and risk factors, like genetics^8^. Similar models have been used to investigate patterns of allergic sensitisation^23–26^, lung function^27,28^ and eczema^29,30^.

Asthma heterogeneity in children has been extensively explored using unsupervised techniques, but most research has relied on wheeze as the central feature for defining and modelling subtypes. While some studies have extended this approach to include additional symptoms such as cough, chest tightness, or breathlessness, the focus has generally remained confined on reported symptoms^31–33^. Parallel work has concentrated on lung function, using spirometry patterns or airway hyperresponsiveness to describe variation^34,35^, yet these physiological measures are often considered in isolation from reported symptoms. Only a small number of studies have attempted to integrate symptomatology with lung function and related markers, leaving a critical gap in our understanding of how clinical manifestations align with physiological and immunological profiles^36–38^. Addressing this gap is essential to move beyond symptom-based categories and towards a more comprehensive characterisation of asthma heterogeneity.

We hypothesise that subgroups of asthma which may better reflect underlying mechanisms can be identified by applying data-driven techniques to detailed information on respiratory symptoms and other key features of asthma (lung function, bronchodilator reversibility, airway hyper-responsiveness and allergic sensitisation), rather than focussing on symptoms alone. Additionally, we propose to integrate the diagnosis of asthma as a key feature to further disaggregate the condition. A clinical diagnosis provides meaningful context, connecting symptoms and physiological traits with a practitioner’s judgement, yet it has rarely been included in clustering approaches. By considering diagnosis alongside symptoms, and objective markers, we capture both the patient-reported experience and the clinical recognition of disease. To do this, we propose to use semi-supervised clustering with Bayesian Profile Regression (BPR), a method that integrates diverse data types while incorporating prior knowledge. By incorporating these elements, the model can capture distinct underlying subgroups, characterised by differences in asthma risk and specific patterns within the pre-defined set of variables.

To test our hypotheses, we performed a multi-domain clustering analysis using data from two population-based birth cohorts. Our analysis incorporated reported symptoms together with several domains central to asthma, including lung function indices (FEV₁/FVC ratio, specific airway resistance before and after bronchodilation), bronchodilator reversibility, airway hyperresponsiveness assessed by methacholine challenge, and allergic sensitisation measured through skin prick tests (SPTs). Finally, we explored early-life and genetic associations, as well as later-life outcomes such as airway inflammation (exhaled nitric oxide, FeNO), to further characterise the derived clusters.

Importantly, asthma diagnosis was included in the clustering model, defined by current wheeze, current medication use, and previous doctor-diagnosed asthma. Since wheeze was already embedded within this definition, it could not be treated as a separate symptom variable without duplicating information and overweighting its influence in the clustering. Therefore, wheeze was excluded from the symptom set to ensure that clusters were derived from distinct and non-overlapping sources of information.

## Methods

Detailed methods and definition of all variables are provided in Supplementary appendix.

### Study design, setting and participants

We used data from two UK population − based birth cohorts. Discovery analysis was carried out in the Manchester Asthma and Allergy Study (MAAS)^39^, and replication in the Isle of Wight Birth Cohort (IOWBC)^40^. Both studies recruited pregnant women who gave birth to 1184 and 1456 children, respectively. Detailed information on the cohorts is provided in the online supplementary material. MAAS study was approved by the North West – Greater Manchester East Research Ethics Committee, while IOWBC obtained ethics approvals from the Isle of Wight Local Research Ethics Committee (now named the National Research Ethics Service, NRES Committee South Central – Southampton B). Informed consent was obtained from all parents, and study subjects gave their assent/consent when appropriate and all experiments were performed in accordance with relevant guidelines and regulations.

### Data sources

*Symptoms and diagnoses*: We used data collected at age 11 years in MAAS and at age 18 in IOW. Validated questionnaires were used to gather data on parent- and self-reported symptoms, physician-diagnosed conditions, and medication use.

*Lung function and bronchodilation reversibility*: At age 11, children in the MAAS study underwent two separate visits. During the first visit, lung function measurements assessed through spirometry (forced expiratory volume in 1 second, FEV₁, and forced vital capacity, FVC) and whole-body plethysmography (specific airway resistance, sRaw) were obtained both before and after bronchodilation (400 mcg salbutamol).

*Airway hyperreactivity*: Children then attended for a second visit, during which a methacholine challenge test was conducted; 610 children completed both visits.

*Airway inflammation*: FeNO was measured as a marker of airway inflammation^41^.

*Allergic sensitisation*: Ascertained using skin prick tests to eight allergens (dog, cat, pollen, mixed grass, house dust mite, mould, eggs, and peanut).

### Variables used to derive clusters

We used 10 variables including the outcome (asthma diagnosis) to derive clusters. Further details on how these variables were derived can be found in the OLS (Table S1).

*Parentally reported symptoms (1–4)*: (1) Shortness of breath; (2) chest tightness, (3) congestion with cold and (4) congestion without cold. The specific questions used to define variables are shown in Table S1.

*Baseline lung function (5)*: z-score for the FEV_1_/FVC GLI % predicted.

*Bronchodilation reversibility (BDR)* (6): Ratio of sRaw before and after bronchodilator use, (7) ratio of FEV_1_ and FVC pre- and post-bronchodilator.

Both lung function and bronchodilation reversibility variables were categorised into eight equal parts based on their distribution, using quantiles (0.125, 0.25, 0.375, 0.5, 0.625, 0.75, 0.875, 1) as cut-offs.

*Airway Hyper-Responsiveness (AHR) (8)*: A *≥* 20% decrease in FEV_1_ by the last dose of the methacholine challenge (16 mg/mL)^11^ (Table S2 for more details).

*Allergic sensitisation (9)*: Defined as a positive SPT (mean wheal diameter of at least 3 mm greater than the negative control to any of allergen). We derived a tailored variable to distinguished between non-sensitised, mono- and poly-sensitised.

*Current asthma (10)*: Defined as a positive answer to two out of three of: ‘*Has the doctor ever told you that your child had asthma*?’, ‘*Has your child had wheezing or whistling in the chest in the last 12 months*?’, and ‘*Has your child had asthma treatment in the last 12 months*?’.

### Objective outcomes used for cluster validation

*Airway inflammation*: FeNO (ppb).

*Early-life risk factors*: Sex, maternal and paternal smoking, parental history of asthma, hay fever, eczema, sibling with asthma, eczema or hay fever, pet ownership before age 5 years. Longitudinal assessment of sensitisation through SPT between age 1 and age 11 was also considered.

*Long term outcome of the derived clusters*: To determine long-term outcomes across clusters in the discovery population, we focused on objective measures from age 8 through early adulthood. This included tracking lung function trajectories, AHR and airway inflammation (FeNO). Data for this analysis were obtained from follow-up assessments conducted at ages 11, 16, and 18–20 years.

### Statistical analysis

#### Asthma risk stratification

To identify distinct profiles, we employed a semi-supervised statistical learning approach. Specifically, we adopted Bayesian Profile Regression (BPR)^42^. Unlike classical regression models, which evaluate the association between variables and outcomes directly, BPR assumes a statistical mixture model. This model groups individuals into clusters, with clusters being jointly determined by both covariates and outcomes. The model is particularly suited when the covariates are highly correlated and when the outcome can also influence the clustering structure. This mixture model comprises an “assignment sub-model”, which assigns individual profiles to clusters, and a “disease sub-model” which connects these clusters to an outcome through a regression model. BPR is based on an Infinite Dirichlet process using a stick-breaking construction and allows the specification of different likelihood structures both for the outcome and the covariates. Given that our outcome is binary (current asthma), we adopted the logit transformation to establish the link between the covariates and the outcome. BPR was implemented in the R package PReMiuM^43^. More information about the model, as well as the choice of the priors’ parameters and the stability checks conducted for the analysis are reported in the OLS (e.g. Figure S1).

#### Risk factors association

Post-hoc analysis using Wilcoxon or Kruskal Wallis test for continuous variables and Fisher exact test or Chi-squared test for categorical was performed to investigate the association between the derived clusters with specific clinical outcomes and early-life risk factors.

#### Genetic association

The 17q12-21 and CDHR3 SNPs selected for this study were chosen due to their previously established associations with childhood-onset asthma, either as lead SNPs or through findings from studies that employed deep phenotyping. Single nucleotide polymorphisms (SNPs) in 17q21, CDHR3 and ANAXA1 were considered^6,18^: “rs7216389”, “rs4795408”,“rs3894194”, “rs6967330”, “rs75260654”, “rs116849664”. The association was evaluated through Fisher test opting for 0.05 significance level.

## Results

### Discovery population: MAAS

Baseline characteristics of the full recruited cohort are presented in Supplementary Table S3. Complete information about the variables used for clustering was available for 500/930 (53.76%) children, who attended age 11 years follow-up. Of those, 100 (20%) had current asthma diagnosis. This subgroup was not statistically different from the excluded observations based on a set of relevant variables (Table S4).

### Derived clusters

In the final clustering allocation, 5 clusters were retrieved (see OLS for models’ details and Figure S2).

**Fig. 1:**
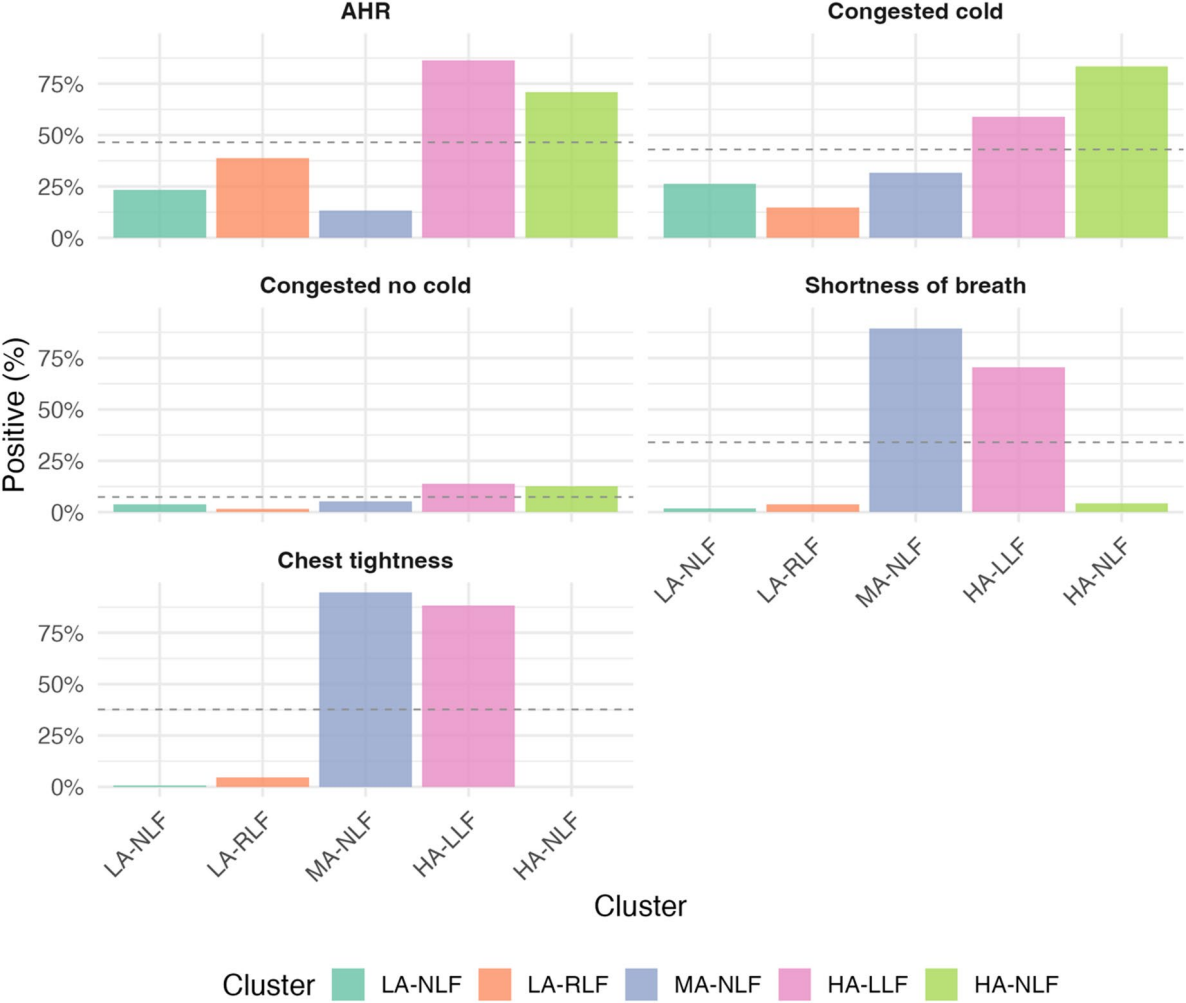
Bayesian profile regression results for MAAS – Reported symptoms and Airway Hyper Responsiveness (AHR). The 5 clusters solution is described along the reported symptoms used in the model and the methacholine challenge results. Bar chart shows represents the percentages of positive answer to the specific question and the percentages of children with a positive methacholine challenge test. In the final clustering allocation, 5 clusters were retrieved formed by 258, 129, 38, 51, and 24 observations of which 9 (3.49%), 6 (4.65%), 20 (52.63%), 41 (80.39%), and 24 (100%) children diagnosed with asthma.

**Fig. 2:**
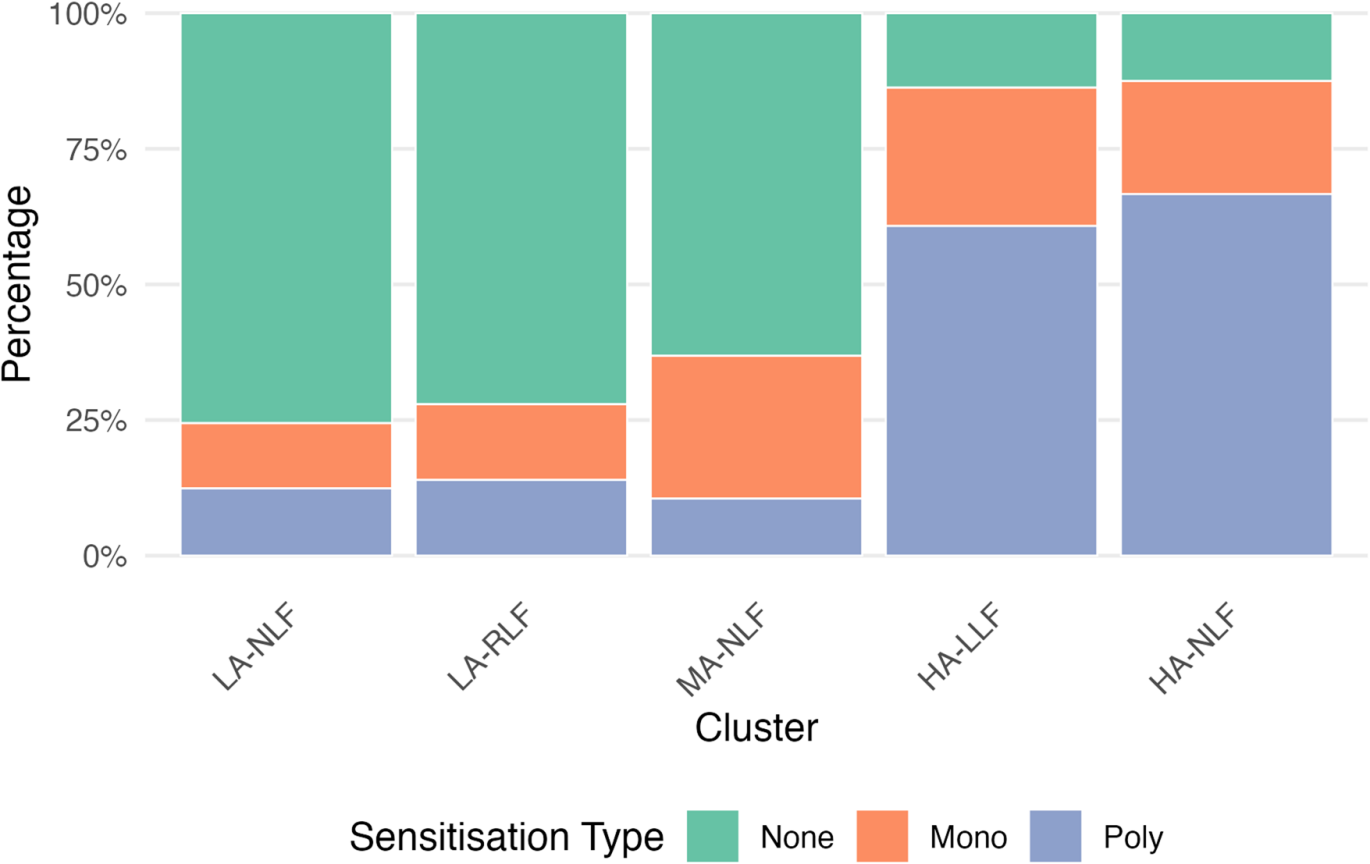
Bayesian profile regression results for MAAS – Sensitisation. The 5 clusters solution is described along the sensitisation type. The stacked bar chart shows the percentage of participants in each cluster classified as non-sensitised (green), mono-sensitised (orange), or poly-sensitised (blue). In the final clustering allocation, 5 clusters were retrieved formed by 258, 129, 38, 51, and 24 observations of which 9 (3.49%), 6 (4.65%), 20 (52.63%), 41 (80.39%), and 24 (100%) children diagnosed with asthma.

**Fig. 3:**
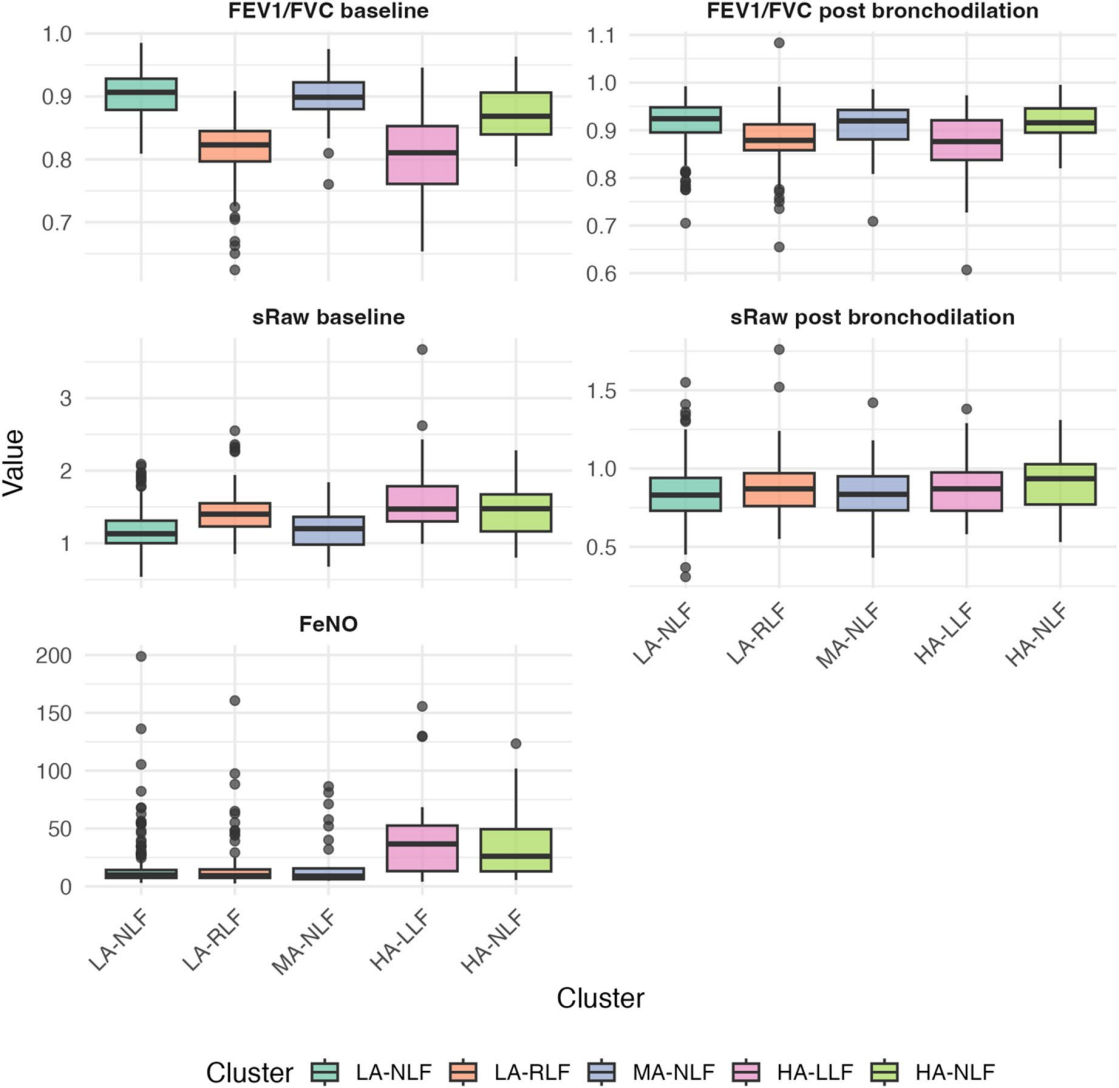
Bayesian profile regression results for MAAS – Lung function measures. The 5 clusters solution is described along some lung functions measures. The solid line represents the median level for each cluster. In the final clustering allocation, 5 clusters were retrieved formed by 258, 129, 38, 51, and 24 observations of which 9 (3.49%), 6 (4.65%), 20 (52.63%), 41 (80.39%), and 24 (100%) children diagnosed with asthma.

*Cluster 1: Low Asthma risk Normal Lung Function (LA-NLF)* (258/500 51.6%) has the lowest probability of reporting any of the symptoms considered in the analysis, a minimal probability of a positive methacholine challenge test (Fig. 1), a low chance of sensitisation (Fig. 2) and normal lung function (Fig. 3). This cluster also has the lowest risk of being diagnosed with asthma (3.49%).

*Cluster 2: Low Asthma risk Reduced Lung Function (LA-RLF)* (129/500 25.8%) despite having a low risk of asthma (4.65%), low probability of a positive methacholine challenge test, or any of the analysed symptoms (Fig. 1) and sensitisation (Fig. 2), children in this cluster exhibited a high probability of reduced lung function and an increased chance of bronchodilator reversibility (Fig. 3).

*Cluster 3 Moderate Asthma risk Normal Lung Function (MA-NLF)* (38/500 7.6%) was associated with a median 50% risk of having asthma diagnosis. This group was characterised by a higher percentage of children reporting shortness of breath or chest tightness upon waking, and a lower percentage of children experiencing congestion in the 12 months preceding the follow-up (Fig. 1). Despite these symptoms, most children in this cluster exhibited normal lung function and BDR and AHR. Finally, they were either non-sensitised or mono-sensitised (Fig. 3).

*Cluster 4 High Asthma risk Low Lung Function (HA-LLF)* (51/500 10.2%) and *Cluster 5 High Asthma risk Normal Lung Function (HA-NLF)* (24/500 4.8%) represented the highest asthma risk profiles, with 80.39% and 100% of children with asthma, respectively. Both clusters were marked by the highest percentages of poly-sensitised children (Fig. 2) who also had positive methacholine test (Fig. 1). In HA-LLF group (Cluster 4), children commonly reported episodes of shortness of breath and chest tightness upon waking before age 8, while these symptoms were not characteristic of children in HA-NLF group (Cluster 5) (Fig. 1). Despite HA-NLF group having the highest percentage of children who were congested with a cold during the 12 months prior to follow-up, the percentage levels of congestion without a cold were similar between HA-NLF and HA-LLF group (Fig. 1). Lung function measures further distinguished these clusters: Cluster HA-LLF consisted of children with poorer lung function with BDR, whereas HA-NLF included children with better baseline lung function who did not exhibit BDR (Fig. 3).

In the HA-NLF cluster, 70.8% of children consistently reported no shortness of breath upon waking at both ages 8 and 11, while 25% developed this symptom between the two time points. A similar pattern was observed for chest tightness, with 75% consistently reporting its absence and 25% reporting its onset during the same period.

Comparison of sRaw before (p-value < 0.001) and after the bronchodilation (*p* = 0.18) in Fig. 3 shows that clusters LA-RLF, HA-NLF and HA-LLF had higher sRaw (i.e. poorer lung function) prior to bronchodilation. However, after treatment, only the HA-NLF cluster retained a median sRaw above the population mean, although this difference did not reach statistical significance.

#### Model robustness and missing data

The model is robust to missing values and no imputation is required. The primary analysis was conducted on the complete data, while the sensitivity analysis, which includes missing values, is detailed in the OLS (Figure S3) showing consistent results with the ones obtained using complete data.

### Characteristics of clusters

#### Airway inflammation and early life risk factors

**Fig. 4:**
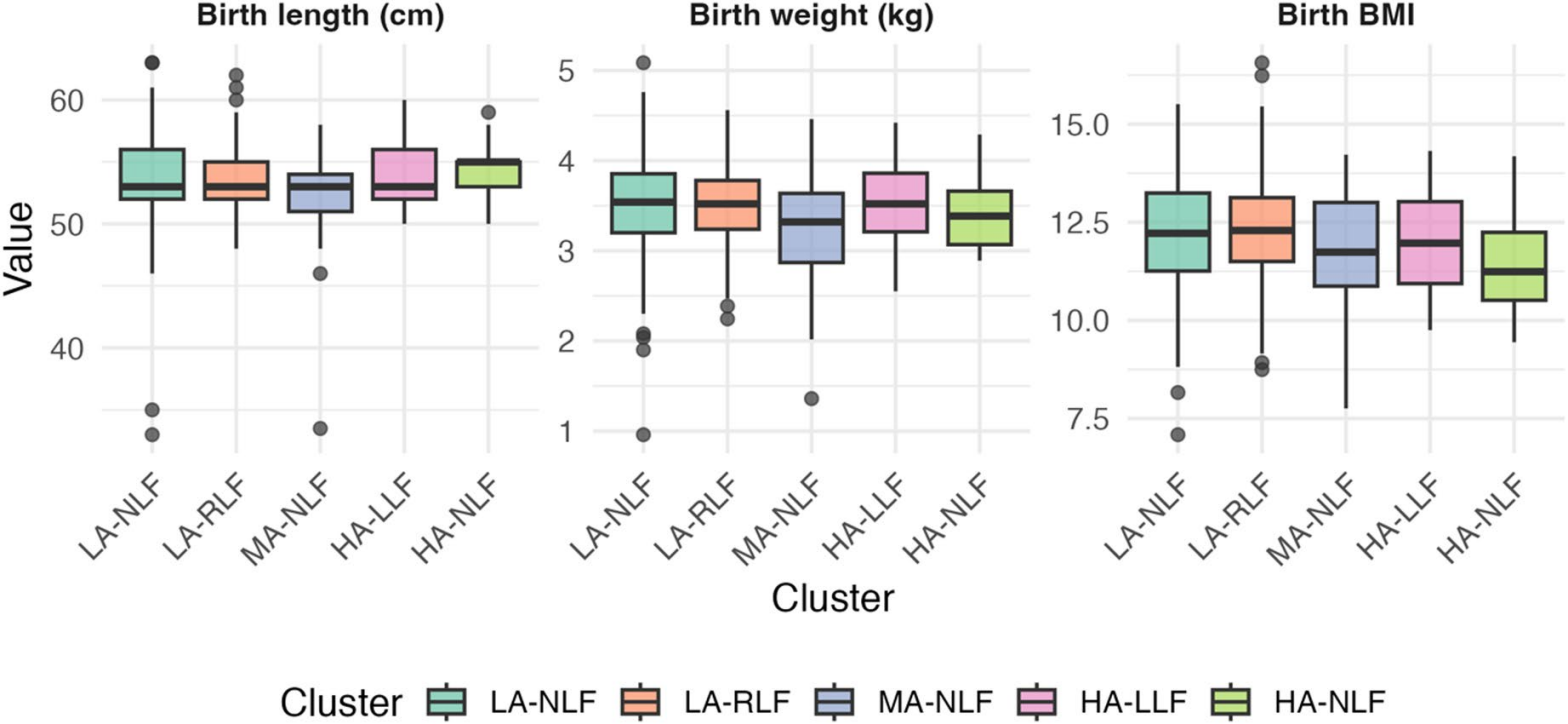
Distribution of birth length, weight, and BMI compared among clusters. The solid line represents the median level for each group.

FeNO levels, revealed higher levels in HA-LLF (Fig. 3). Details regarding the child’s birth length, weight, and BMI are provided in Fig. 4. Although the overall test for BMI suggested a marginal association (*p* = 0.075), the comparison between children in HA-NLF and the rest of the population revealed a statistically significant difference (*p* = 0.017), indicating that children in HA-NLF had lower BMI.

Associations with early risk factors are summarised in Table 1. While the only parental characteristic that has a significantly different distribution among the clusters is maternal eczema, all the risk factors involving the sibling have significantly different distributions, with MA-NLF and HA-NLF group having the highest percentages of siblings with eczema and hay-fever.

**Table 1:** Risk factors distribution among the retrieved clusters and simulated p-value of fisher exact test.

	LA-NLF *n*/*N* (%)	LA-RLF *n*/*N* (%)	MA-NLF *n*/*N* (%)	HA-LLF *n*/*N* (%)	HA-NLF *n*/*N* (%)	*p*-value
Mode of delivery (vaginal)	175/218 (80.28)	76/105 (72.38)	26/34 (76.47)	39/45 (86.67)	20/24 (83.33)	0.308
Maternal smoking (yes)	25/258 (9.69)	13/129 (10.08)	8/38 (21.05)	4/50 (8.00)	2/24 (8.33)	0.325
Maternal asthma ever (yes)	46/258 (17.83)	26/129 (20.16)	8/38 (21.05)	12/51 (23.53)	6/24 (25.00)	0.761
Maternal current asthma (yes)	33/258 (12.79)	19/129 (14.73)	5/38 (13.16)	8/51 (15.69)	6/24 (25.00)	0.551
Maternal hay-fever (yes)	68/257 (26.46)	31/129 (24.03)	10/38 (26.32)	19/51 (37.26)	9/24 (37.50)	0.326
Maternal eczema (yes)	39/258 (15.12)	17/129 (13.18)	12/38 (31.58)	7/51 (13.73)	9/24 (37.50)	**0.007**
Paternal smoking (yes)	61/258 (23.64)	32/129 (24.81)	11/38 (28.95)	9/50 (18.00)	5/24 (20.83)	0.805
Paternal asthma ever (yes)	32/258 (12.40)	22/129 (17.05)	5/38 (13.16)	9/51 (17.65)	6/24 (25.00)	0.356
Paternal current asthma (yes)	17/258 (6.59)	13/129 (10.08)	4/38 (10.53)	6/51 (11.76)	3/24 (12.50)	0.423
Paternal hay fever (yes)	57/258 (22.09)	35/129 (27.13)	7/38 (18.42)	10/51 (19.61)	5/24 (20.83)	0.732
Paternal eczema (yes)	24/258 (9.30)	12/129 (9.30)	4/38 (10.53)	7/51 (13.73)	5/24 (20.83)	0.387
Older sibling with asthma (yes)	25/258 (9.69)	7/129 (5.43)	6/38 (15.79)	9/50 (18.00)	6/24 (25.00)	**0.009**
Older sibling with eczema (yes)	30/258 (11.63)	8/129 (6.2)	11/38 (28.95)	8/50 (16.00)	6/24 (25.00)	**0.001**
Older sibling hay-fever (yes)	9/258 (3.49)	0/129 (0.00)	4/38 (10.53)	3/50 (6.00)	3/24 (12.50)	**0.001**
Ever pet owner by age 5	105/251 (41.83)	53/123 (43.09)	18/35 (51.43)	11/38 (28.95)	5/22 (22.73)	0.127
Sex (female)	132/258 (51.16)	66/129 (51.16)	17/38 (44.74)	19/51 (37.25)	8/24 (33.33)	0.194

The evolution of sensitisation from the first year of life to age 11 shows consistently high percentages of sensitised children in groups HA-NLF and HA-LLF throughout most of the period considered (Figure S4).

#### Genetic associations

The genetic analysis (Table 2) showed no statistically significant associations CDHR3-related SNPs and the clusters. In contrast, a significant association was observed for the 17q21 SNP rs3894194 (p-value = 0.005), with the asthma associated variant being less frequent in Cluster 4 (HA-LLF), despite the high risk of asthma, in contrast to the other high asthma risk cluster, cluster 5.

**Table 2:** Distribution of genetic variations among the retrieved clusters.

SNPs			LA-NLF	LA-RLF	MA-NLF	HA-LLF	HA-NLF	*p*-value
			*N* = 252	*N* = 126	*N* = 38	*N* = 50	*N* = 24	
17q21 and CDHR3	rs7216389	CC	27.38%	28.57%	21.05%	30.00%	16.67%	0.907
		CT	48.02%	42.86%	50.00%	46.00%	54.17%	
		TT	24.60%	28.57%	28.95%	24.00%	29.17%	
	rs4795408	GG	34.52%	39.68%	18.42%	28.00%	25.00%	**0.050**
		GA	42.46%	38.89%	65.79%	60.00%	50.00%	
		AA	23.02%	21.43%	15.79%	12.00%	25.00%	
	rs3894194	GG	34.13%	41.27%	18.42%	28.00%	25.00%	**0.005**
		GA	43.25%	38.10%	63.16%	66.00%	50.00%	
		AA	22.62%	20.63%	18.42%	6.00%	25.00%	
	rs6967330	GG	68.65%	75.40%	60.53%	60.00%	70.83%	0.305
		GA	29.37%	21.43%	36.84%	34.00%	29.17%	
		AA	1.98%	3.17%	2.63%	6.00%	0.00%	
			*N* = 241	*N* = 121	*N* = 37	*N* = 47	*N* = 23	
ANAXA1	rs75260654	CC	97.93%	93.39%	91.89%	93.62%	100%	0.067
		TC	2.07%	6.61%	8.11%	6.38%	0%	
		TT	0%	0%	0%	0%	0%	
	rs116849664	CC	98.34%	95.87%	91.89%	93.62%	100%	0.083
		TC	1.66%	4.13%	8.11%	6.38%	0%	
		CC	0%	0%	0%	0%	0%	

#### Long term outcome

The analysis of long-term outcomes shows that HA-LLF is characterised by the poorest lung function and a higher incidence of reported wheezing. In contrast, HA-NLF shows elevated FeNO levels and a high frequency of reported symptoms, despite having a normal FEV_1_/FVC z-score. The distinction between LA-NLF and LA-RLF over time is mainly driven by differences in their FEV_1_/FVC z-scores (Figure S5-c).

The proportion of positive methacholine challenge tests within each cluster at various ages (8, 11, 18) is reported in Figure S5a showing consistent differences across time, with clusters HA-NLF and HA-LLF consistently displaying the highest percentages.

### Validation population: IOW

To validate the results, we repeated the analysis using the data collected in the IOWBC for young adults at age 18. Due to partial overlap between the two questionnaires, it was not possible to include the reported symptoms, and only the available objective measures were used.

For the methacholine challenge if in at least one of the 9 steps conducted during the, the FEV_1_ was less than 80% of the baseline level, we considered the test as positive. We derived the z-score, the ratio pre-post, and categorise them according to the quantile distribute on described before.

The sensitisation status was derived based on 6 out of 8 overlapping allergens utilised for MAAS (dog, cat, egg, tree, mixed grass, peanut).

We analysed data from 516 participants with complete data, of whom 103 (19.96%) had asthma diagnosis; resulting in a very similar sample size and outcome distribution for the two data set considered.

**Fig. 5:**
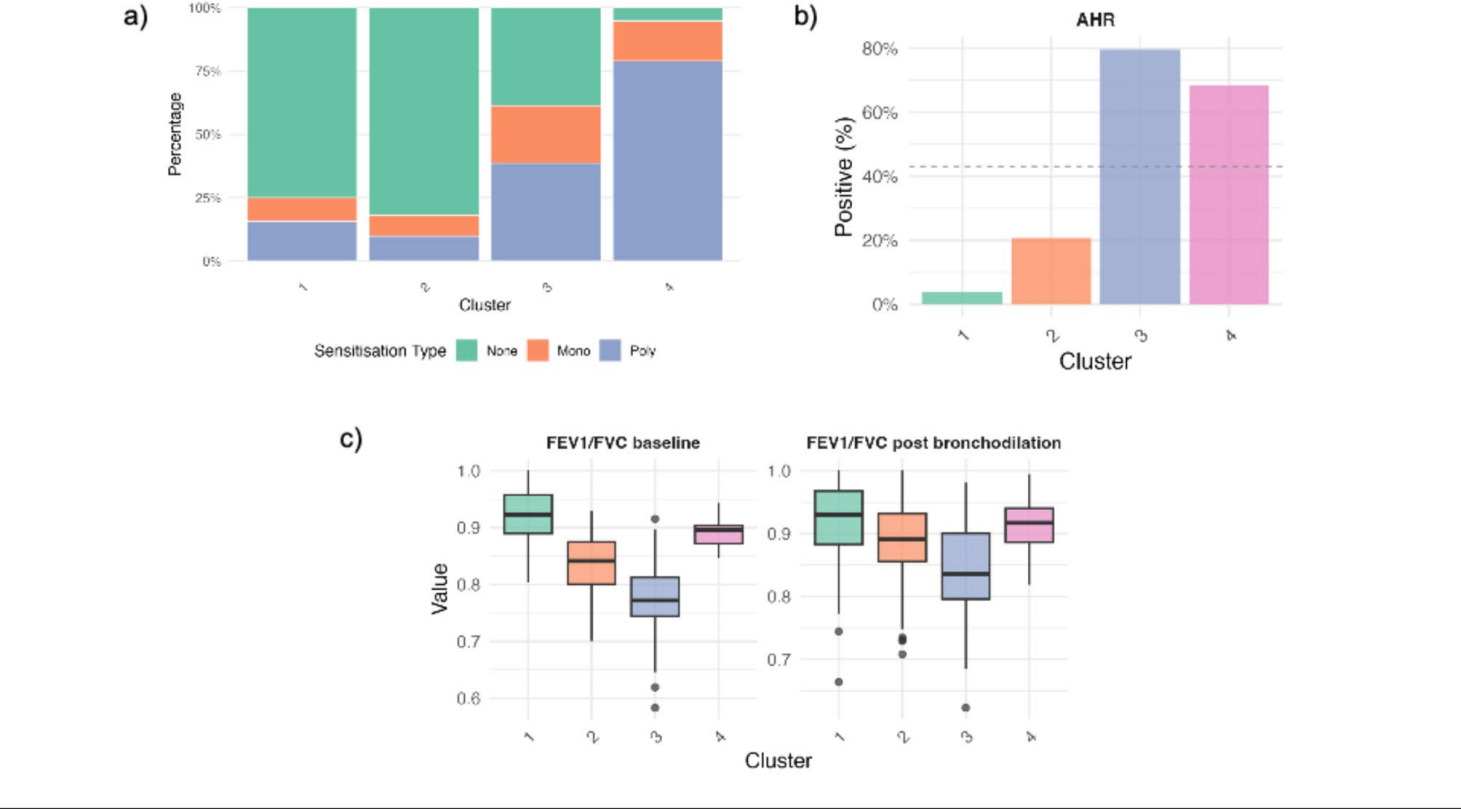
Bayesian profile regression results for IOW – cluster characteristics. (**a**) Distribution of allergen sensitisation patterns within each cluster. Bars display the percentage of participants who were non-sensitised (green), mono-sensitised (orange), or poly-sensitised (blue). (**b**) Proportion of individuals with airway hyperresponsiveness (AHR) in each cluster, expressed as percentage positive. (**c**) Boxplots showing FEV₁/FVC values at baseline (left) and post-bronchodilation (right) across clusters.

Without the information about the reported symptoms, only 4 groups were identified by the model. These clusters closely mirrored those described earlier in terms of size and the percentage of subjects with asthma (Figs. 5 and S6). LA-NLF primarily consisted of individuals with good lung function, no sensitisation, negative methacholine tests, and no bronchodilator responsiveness. This group had the lowest risk of having asthma. In contrast, subjects in the LA-RLF were characterised by lower lung function and higher broncho-responsiveness. Additionally, the model identified two groups with a high risk of asthma diagnosis. As for the MAAS profiles, both groups were characterised by a high sensitisation rate and positive methacholine challenge tests. However, they differ in baseline lung function and bronchodilator response.

## Discussion

By employing a Bayesian Profile Regression model on comprehensive information on airway hyperresponsiveness, reported symptoms, bronchodilation reversibility, lung function, sensitisation and asthma diagnosis in two population-based birth cohorts we have identified five clusters at population level with distinct asthma risk profiles.

Clusters 1 and 2 (LA-NLF and LA-RLF) exhibited the lowest asthma risks, with minimal symptoms and negative methacholine test. Despite a low risk of asthma, LA-RLF is characterised by diminished lung function and a greater propensity for bronchodilator reversibility. One possible explanation is that children in this cluster, starting with lower baseline lung function, had more potential for improvement after receiving 400 mcg of salbutamol. The absence of any significant differences in lung function after bronchodilator use would indicate that this group does not exhibit characteristics of fixed airway obstruction. Interestingly, this cluster demonstrates that impaired lung function and bronchodilator responsiveness can occur without asthma symptoms, suggesting a subgroup with subclinical changes or alternative causes of reversibility. Importantly, it shows that BDR on its own is not sufficient for diagnosis and that symptom assessment and clinical context remain crucial.

In contrast, Clusters 4 and 5 (HA-LLF, HA-NLF) represented the highest asthma risk profiles, with significant percentages of poly-sensitised children and abnormal lung function measures. Notably, HA-LLF group had the highest median sRaw levels, indicating more severe airway obstruction. Cluster 3 (MA-NLF), associated with a moderate risk of asthma, was characterised by specific symptom patterns such as shortness of breath and chest tightness upon waking. Despite these symptoms, children in MA-NLF group generally exhibited normal lung function and BDR, differentiating them from the higher-risk clusters.

The analysis of early-life and genetic associations improved the characterisation of the identified clusters. Association with Exhaled Nitric Oxide (FeNO) levels and with genetic variants of 17q21 SNP rs3894194, were described, particularly affecting MA-NLF, HA-NLF, and HA-LLF.

Validation with an independent cohort corroborated our findings, identifying similar asthma risk profiles. This validation highlights the reliability of our model and the consistency of the identified subgroups across different datasets.

One limitation of the study is differences between the two birth cohorts used in age of assessment and the available information. However, the results obtained in both cohorts are consistent. The technical limitation of the model concerns the difficulties in assessing the convergence of the algorithm^43,44^. Nonetheless, previous work has outlined practical strategies to detect situations where convergence has not been achieved^44^. We performed several controls in this direction, and the model parameters were accurately selected to achieve satisfactory results in terms of stability. Additional information is added in the OLS.

While earlier studies largely focused on wheeze phenotypes or lung function separately^6,7,45,46^, our findings highlight that asthma subgroups defined through holistic, multi-domain clustering provide a more detail overview of disease heterogeneity^36,47^. The five derived clusters captured distinct asthma risk profiles, revealing important variations in lung function, sensitisation, and response to bronchodilators. Notably, clusters with low asthma risk exhibited either normal or reduced lung function (LA-NLF and LA-RLF), but the latter demonstrated increased BDR. In contrast, moderate-to-high asthma risk clusters (MA-NLF, HA-NLF, HA-LLF) were distinguished by differences in airway inflammation, sensitisation, and AHR, reinforcing prior observations that asthma subgroups vary not only in symptom patterns but also in biological markers. Importantly, while previous studies have noted variability in wheeze retrieved clusters in relation to symptom patterns, prevalence, and associated risk factors^19,20^, our approach assumes that asthma risk can be stratified based on comprehensive clinical and physiological profiles rather than symptoms alone trying to include the risk of being diagnosed with asthma as important feature to derive the clusters.

Furthermore, our findings validate the importance of airway hyperresponsiveness and sensitisation as key drivers of asthma heterogeneity, with HA-LLF and HA-NLF clusters displaying the highest rates of poly-sensitisation and positive methacholine test results. The genetic associations observed, particularly with 17q21 SNP rs3894194 mapping to GSDMA, align with prior research linking this locus to asthma susceptibility^48–51^. Although the specific mechanisms involved remain to be elucidated, this alignment with established asthma genetics supports the biological relevance of variation across our clusters. Interestingly, the asthma-associated allele was less frequent in Cluster 4 (HA-LLF), despite this group’s elevated asthma risk. In contrast, Cluster 5, which also exhibited high asthma risk, showed the expected increased prevalence of the 17q21 risk allele. This divergence between two clinically high-risk clusters highlights the heterogeneity of asthma pathophysiology, highlighting how similar phenotypic risk profiles arise from distinct mechanisms. Additionally, long-term outcome analysis indicates that high-risk clusters maintain distinct lung function trajectories and symptom burdens over time, emphasizing the clinical relevance of these classifications.

Overall, our study underscores the need for holistic, multi-domain clustering approaches in asthma research^47^. By incorporating diverse clinical and biological markers, our methodology advances the precision of asthma subtyping and provides a framework for future personalised treatment strategies.

In conclusion, our study highlights the utility of integrating diverse data sources and advanced statistical methods to investigate asthma subtypes. By combining patient-reported symptoms with objective measures, we provide a more comprehensive understanding of asthma heterogeneity. Further studies are needed to explore the implications of these findings for targeted interventions and to validate the clinical relevance of the identified sub-groups.

## Supplementary Information

Below is the link to the electronic supplementary material.

Supplementary Material 1

## Data Availability

Summary statistics are available from the corresponding author upon request.
