# Supplementary material for: Clustering lung function and symptom profiles for asthma risk stratification
Source: Sci Rep. 2025 Dec 24;16:3110. doi: 10.1038/s41598-025-32977-w (PMC12830796; doi:10.1038/s41598-025-32977-w)
Supplement: Supplementary file 1 — Supplementary Material 1 [file 41598_2025_32977_MOESM1_ESM.docx]

**Clustering lung function and symptom profiles for asthma risk stratification**

Alex Cucco^1,2^, Angela Simpson^3^, Clare Murray^3^, Graham C Roberts^4,5,6,7^, John W Holloway^5,7^, S. Hasan Arshad^4,5,6^, Adnan Custovic^†1^, Sara Fontanella^†1^

^1^National Heart and Lung Institute, Imperial College London, UK

^2^ Department of Socio-Economic, Managerial, and Statistical Studies, University “G. d’Annunzio” of Chieti-Pescara, Pescara, Italy

^3^Division of Infection, Immunity and Respiratory Medicine, School of Biological Sciences, Faculty of Biology, Medicine and Health, University of Manchester, Manchester Academic Health Science Centre, UK

^4^Clinical and Experimental Sciences, Faculty of Medicine, University of Southampton, Southampton, UK;

^5^NIHR Southampton Biomedical Research Centre, University Hospital Southampton, Southampton, UK;

^6^David Hide Asthma and Allergy Centre, St Mary's Hospital, Isle of Wight, UK.

^7^Human Development & Health, Faculty of Medicine, University of Southampton, Southampton SO17 1BJ, UK

^†^Joint senior authors

**ONLINE SUPPLEMENTARY MATERIAL**

**DATA SOURCES: DESCRIPTION OF COHORTS**

*The Manchester Asthma and Allergy Study (MAAS)*

MAAS is an unselected birth cohort study established in 1995 in Manchester, UK (11). It consists of a mixed urban-rural population within 50 square miles of South Manchester and Cheshire, located within the maternity catchment area of Wythenshawe and Stepping Hill Hospitals. All pregnant women were screened for eligibility at antenatal visits (8-10th week of pregnancy). Of the 1499 couples who met the inclusion criteria (≤10 weeks of pregnancy, maternal age ≥18 years, and questionnaire and skin prick data test available for both parents), 288 declined to take part in the study and 27 were lost to follow-up between recruitment and the birth of a child. A total of 1184 children were born into the study between February 1996 and April 1998. They were followed prospectively for 20 years to date and attended follow-up clinics for assessments, which included lung function measurements, skin prick testing, biological samples (serum, plasma and urine), and questionnaire data collection. The study was approved by the North West – Greater Manchester East Research Ethics Committee.

*The Isle of Wight Birth Cohort (IOWBC)*

IOW is an unselected birth cohort study established in 1989 on the Isle of Wight, UK (13-15). After the exclusion of adoptions, perinatal deaths, and refusal for follow-up, written informed consent was obtained from parents to enrol 1,456 newborns (of 1536 born between 1^st^ January 1989 and 28^th^ February 1990). Follow-up-up assessments were conducted to 26 years of age to prospectively study the development of asthma and allergic diseases. At each follow-up, validated questionnaires were completed by the parents. At 10 years, spirometry was performed as described below. Ethics approvals were obtained from the Isle of Wight Local Research Ethics Committee (now named the National Research Ethics Service, NRES Committee South Central – Southampton B) at recruitment and for the subsequent follow-ups.

**Lung Function Measurement**

In the MAAS study, spirometry was conducted following the American Thoracic Society/European Respiratory Society guidelines using a Lilly pneumotachograph system with animated incentive software (Jaeger, Germany). For home visits, a flow turbine spirometer (Micro Medical, UK) was used. Participants were instructed to inhale to total lung capacity (TLC) and then perform a forced expiration through a mouthpiece until reaching residual volume (RV). The test was repeated at 30-second intervals until three technically acceptable traces were obtained. Forced expiratory volume in one second (FEV₁) and forced vital capacity (FVC) were recorded, with results expressed as FEV₁ % predicted and the FEV₁/FVC ratio.

Specific airway resistance (sRaw) was measured in all children who were willing to cooperate. A constant volume whole-body plethysmograph (Masterscreen Body 4.34; Jaeger, Würzburg, Germany) was used to measure sRaw through a single-step procedure, derived from simultaneous recordings of respiratory flow and plethysmographic pressure changes, without measuring thoracic gas volume (TGV). Measurements were performed during normal tidal breathing using a modified facemask (Astratech No. 2; Astra, Denmark) fitted with a non-compressible mouthpiece. Once a stable breathing pattern was established, at least three sRaw measurements were taken, each calculated from the mean of five consecutively recorded, technically acceptable loops. The median of these three effective sRaw measurements was used for analysis. Values were corrected for the influence of the pneumotachograph screen and volume displacement caused by the subject.

**IOWBC**

Lung function tests were performed before bronchodilator administration at age, 18. Forced vital capacity (FVC) and forced expiratory volume in 1 second (FEV1) were measured using a Koko Spirometer and software with a portable desktop device (both from PDS Instrumentation, Louisville, KY, USA). Spirometry was conducted and assessed following the American Thoracic Society (ATS) guidelines. Participants, whether children or adults, were required to be free from respiratory infections for two weeks, not using oral steroids, and instructed to refrain from β-agonist medications for 6 hours and caffeine for at least 4 hours prior to testing.

**Allergic sensitisation**

Skin prick testing to a battery of allergens including dust mite, tree, grass and weed pollens, moulds, cat, dog, cockroach, and horse was conducted. Weal 3 mm greater than negative control was considered a positive reaction.

**Methacholine Challenge**

Airway reactivity was assessed using a methacholine challenge following a 5-step protocol in accordance with American Thoracic Society guidelines. Participants inhaled quadrupling doses of methacholine (0.0625–16.0 mg/mL) via a DeVilbiss 646 nebulizer (Sunrise Medical HHG, Somerset, PA) and a KoKo dosimeter (Pulmonary Data Services, Doylestown, PA), calibrated to deliver 0.009 mL per 0.6-second actuation. FEV₁ was predicted, and if the measured value was below 1.0 L or less than 60% of the predicted value, the test was not conducted. FEV₁ was recorded at 30 and 90 seconds following five inhalations of each methacholine dose. The challenge was terminated either upon detecting a 20% reduction in FEV₁ or after administering the highest methacholine concentration.

**FeNO**

In the MAAS study, fractional exhaled nitric oxide (FeNO) was measured using a chemiluminescence analyzer (NIOX, Aerocrine, Sweden) or an electrochemical analyzer (NIOX Mino, Aerocrine, Sweden). The device was changed on May 4, 2012, but previous studies confirmed that both analyzers provided comparable results.

**METHODS: Bayesian Profile Regression**

*Model description*

Bayesian profile regression^1-6^ is a Bayesian model that allows connecting a response variable to a set of covariates, via a clustering structure. This model is particularly suited in the case of highly correlated features. The outcome variable can be continuous, as well as discrete, and the covariates can as well be a set of continuous and/or categorical variables. Although, using a mixed set of covariates requires an independence assumption between continuous and categorical variables. In the case of a binary outcome, a logit link is used. The model uses a Dirichlet prior via a stick-breaking construction for the parameters of an infinite mixture model specified based on the distribution of both the outcome and the covariates. As it uses an infinite mixture model with a stick-breaking construction, the sampler used to approximate the posterior distribution should allow a label switching. While usually, the efforts are towards avoiding labels switching during the sampling procedure, in the case of a stick-breaking construction, the prior is not independent on the clustering order^1,2,7^, and consequently, the label switching is forced for convergence reasons. As the number of clusters, as well as the clustering labels, can change between different sweeps of the sampler, the final clustering allocation is retrieved summarising all the information obtained during the sampling procedure. For each sweep, if a pair of observations is allocated to the same cluster, a value of 1 is assigned to this couple, otherwise a value of 0. This procedure is iterated over all sweeps, and a similarity measure is retrieved averaging by the total number of iterations. The similarity measure is then processed via the PAM algorithm.

*Model priors and parameters*

To select the model’s parameters, we followed the instruction specified in ^1,2^. After running models using 3 different priors for the parameter α, we compared the final solutions as well as the convergence of the sampler using three different initial numbers of clusters and three different seeds. We opt for the prior specified by the authors as the default prior (Gamma(2,1)). The final clustering allocation was robust using different prior specifications. Given the prior specification, we evaluated the log marginal posterior^1^ using different numbers of clusters for the initial allocation and compared the solutions using three different seeds. The solution was stable concerning the seed modification, and 60 initial clusters were utilised to initialise the algorithm. This choice allows exploring higher parameter space, with the drawback of a longer sampling procedure. We used the ‘SliceIndipendet’ sampler described in^1^ with the default label-switching moves. The results are based on the 50000 final iterations of the sampler after an initial burn-in of 50000 iterations. The impact of the categorisation of the continuous variables, as well as the categorisation of the sensitisation type, were tested using different cut points and using a binary classification in ‘sensitised’ and ‘not sensitised’. Also, in this case the clustering allocation was robust.

**Table S1 Detailed information used to cluster**

| **MAAS** | *In the last 12 months has your child usually seemed congested in the chest or coughed up phlegm (mucus) when she does not have a cold?* |
| --- | --- |
|  | *In the last 12 months has your child usually seemed congested in the chest or coughed up phlegm (mucus) with colds?* |
|  | *woken with chest tightness ever till age 8 (previous follow up)* |
|  | *woken with SOB ever till age 8 (previous follow up)* |
|  | *fev1/fvc zscore from GLI* |
|  | *Ratio pre/post broncho dilation (of the fev1/fvc ratio)* |
|  | *ratio sraw pre/post broncho dilation dilation* |
|  | *methacholine - positive vs negative* |
|  | *Non-sensitised, mono sensitised, polisensitised SPT considered: cat, dog, egg, mite, mold, peanut, pollen, tree* |
| **IOW** | *fev1/fvc zscore from GLI* |
|  | *Ratio pre/post broncho dilation (of the fev1/fvc ratio)* |
|  | *methacholine - positive vs negative* |
|  | *Non-sensitised, mono sensitised, polisensitised SPT considered: dog, cat, tree, egg, peanut, grass* |

**Methacholine challenge**

During the methacholine test, the challenge was halted before administering the final dose for 9 of the children. However, as shown in Table S2, their FEV1 levels had already dropped significantly at lower doses. Due to this considerable decline, these children were still classified as reactive to the challenge.

**Table S2. Methacholine challenge description for 9 children**

|  | **Last_dose** | **FEV1 last dose / BaselineFEV1** | **Methacholine**  **Test results** |
| --- | --- | --- | --- |
| **Subject 1** | ‘Not received’ | 80.56% | ‘reactive’ |
| **Subject 2** | ‘Not received’ | 81.08% | ‘reactive’ |
| **Subject 3** | ‘Not received’ | 81.08% | ‘reactive’ |
| **Subject 4** | ‘Not received’ | 80.36% | ‘reactive’ |
| **Subject 5** | ‘Not received’ | 80.77% | ‘reactive’ |
| **Subject 6** | ‘Not received’ | 80.43% | ‘reactive’ |
| **Subject 7** | ‘Not received’ | 80.95% | ‘reactive’ |
| **Subject 8** | ‘Not received’ | 80.56% | ‘reactive’ |
| **Subject 9** | ‘Not received’ | 81.08% | ‘reactive’ |

**Table S3. Demographic characteristics of the study cohorts.**

Continuous variables are presented as mean ± standard deviation (SD). Categorical variables are presented as n/N (%), where n is the number of participants with the characteristic and N is the number with available data.

|  | **MAAS** | **IOW** |
| --- | --- | --- |
| *Continuous - mean (sd)* | | |
| Gestational Age | 39.86(1.61) | 39.87(1.62) |
| Maternal age | 30.38(4.78) | 26.77(5.36) |
| Breast feeding Week | 15.46(20) | 14.07(14.61) |
| BMI | 12.14(1.7) | 343.61(123.9) |
| *Binary - n/N (%)* | | |
| Sex (male) | 648/1184(54.73%) | 786/1536(51.12%) |
| Maternal smoking | 174/1184(14.7%) | 165/1525(10.82%) |
| Maternal asthma | 235/1184(19.85%) | 165/1525(10.82%) |
| Paternal smoking | 312/1184(26.35%) | 393/1517(25.91%) |
| Paternal asthma | 163/1182(13.79%) | 608/1511(40.24%) |

**Table S4. Comparison of included and excluded subjects in MAAS.**

|  | **Subjects with complete data** | **Subjects with incomplete data** | **p-value** |
| --- | --- | --- | --- |
| **n** | 500 | 430 |  |
| **Sex (F)** | 48.40% | 42.79% | 0.10 |
| **Maternal asthma ever** | 19.60% | 20.47% | 0.74 |
| **Maternal current asthma at recruitment** | 14.20% | 13.42% | 0.44 |
| **Mother hay fever** | 27.45% | 27.67% | 0.97 |
| **Mother eczma** | 16.80% | 16.28% | 0.86 |
| **Delivery type** | 78.87% | 80.52% | 0.59 |
| **Asthma derived** | 20.00% | 20.93% | 0.57 |

**Stability check in Bayesian Profile Regression**

To evaluate the robustness of the derived clusters with respect to different choices of prior specification for the parameter α, we compared the solution retrieved using three different set of parameters: Gamma (3; 3), Gamma (2; 1) and Gamma (3.5; 1).

Despite different prior specification, the cluster solutions were stable. The retrieved allocation is identical using the 3 priors, with only a child changing allocation. The default prior (Gamma 2,1) was selected for the analysis.

To check the stability of the clustering solution, as well as validate the convergence of the algorithm, we evaluated the log-marginal model posterior using a different number of clusters to initialise the algorithm^1^ (Figure S1) using the selected prior. The solution proposed is reasonably stable and from Figure S1, no clear signal of non-convergence of the algorithm is highlighted. Stable results were also identified using the other two priors.

We also checked the stability of the results varying seeds and using different categorisation for the continuous variables as well as for the sensitisation variable. All the replications lead to the identification of 5 clusters with identical profiles.

The model was run using MCMC, with a total of 100000 iterations after excluding the initial 50000 iterations for burn-in.

**Figure S1. Log marginal model posterior using a different number of clusters to initialise the algorithm.**


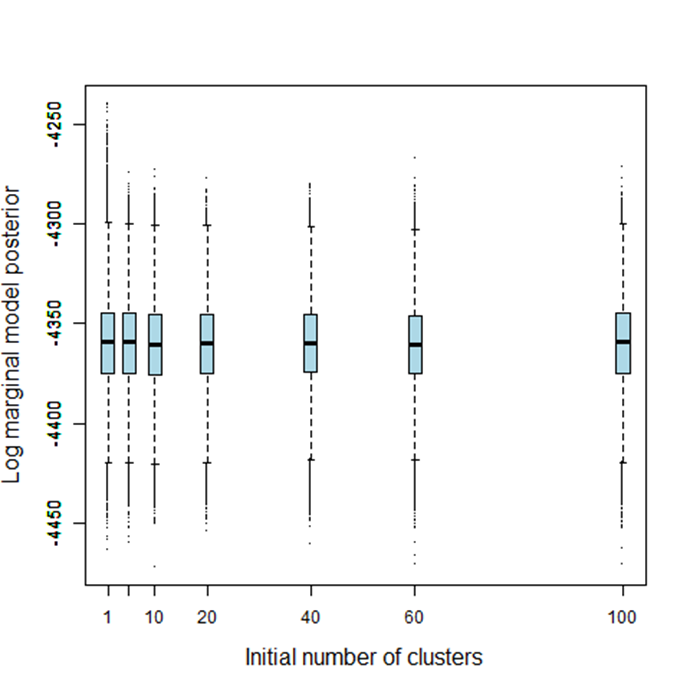


**Figure S2. Posterior distributions of asthma-related parameters and covariates across derived clusters.**

Box plot colours indicate the posterior deviation from the overall mean estimate: red denotes significantly higher values, green denotes values close to the mean, and blue denotes significantly lower values. For covariates, colours reflect whether the posterior probability distribution is significantly above (red), similar to (green), or below (blue) the expected value. Colours indicate statistical deviation only and do not correspond to cluster membership.


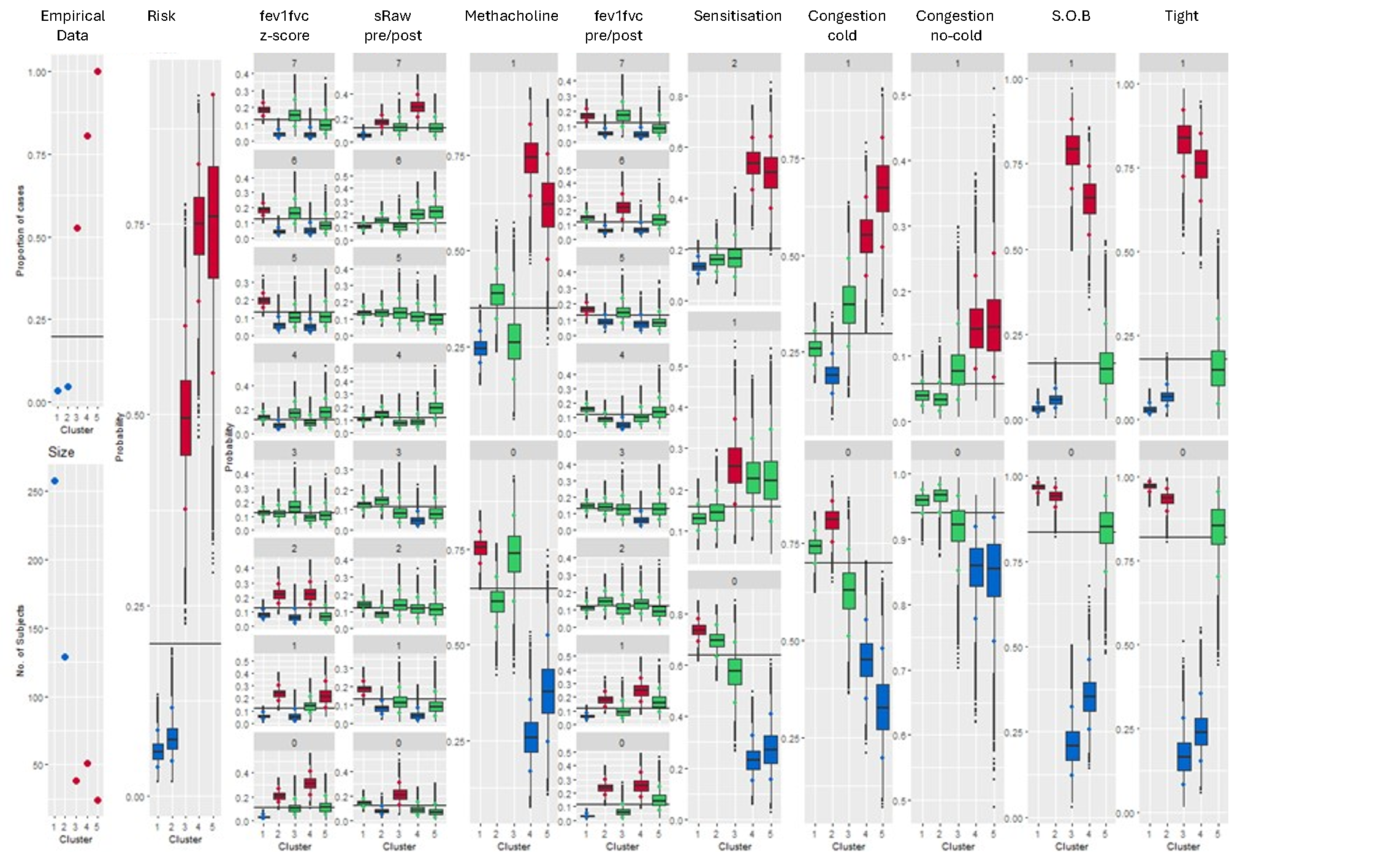


**Analysis with missing values**

The 5 clusters solution is described along the variables used in the model.

**Figure S3. Bayesian Profile Regression applied to the complete dataset including observations with missing data to assess robustness of the cluster structure.**

Box plot colours indicate the posterior deviation from the overall mean estimate: red denotes significantly higher values, green denotes values close to the mean, and blue denotes significantly lower values. For covariates, colours reflect whether the posterior probability distribution is significantly above (red), similar to (green), or below (blue) the expected value. Colours indicate statistical deviation only and do not correspond to cluster membership.


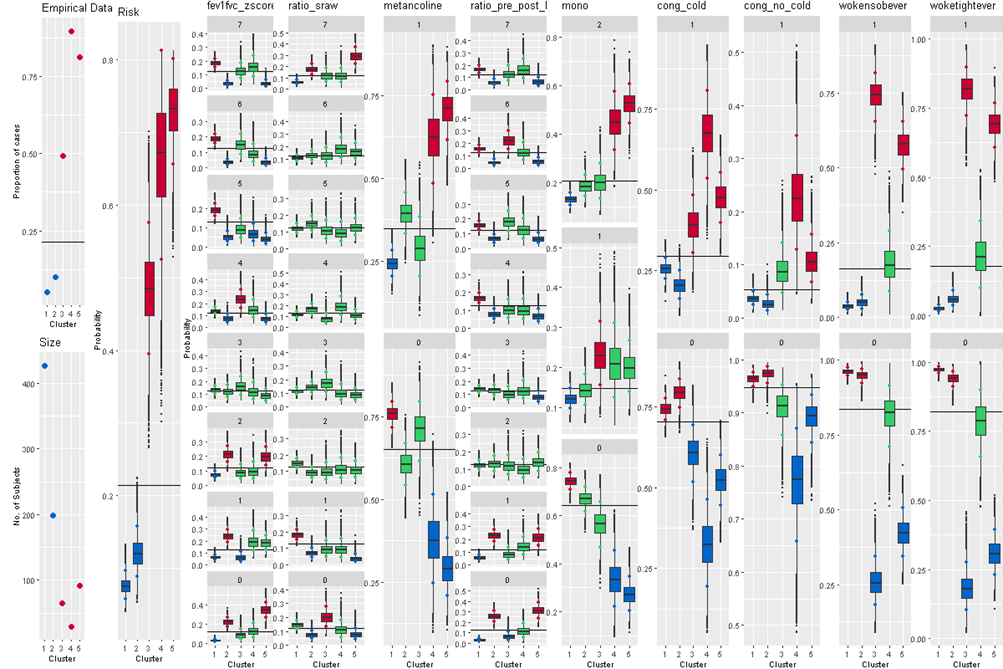


**SENSITISATION – MAAS**

**Figure S4. Sensitisations for each cluster from age 1 to age 11.**

The number of skin prick test results available at each time point is depicted in the table, stratified by cluster. Different colours are used for different clusters, the median level for each cluster is displayed . Light green = cluster 1, dark green = cluster 2, yellow = cluster 3, red = cluster 4, orange = cluster 5.

**Figure S5. Longitudinal trajectories of clinical characteristics across clusters from age 8 to 18 years.**

a) *Proportion of positive methacholine challenge for each cluster from age 8 to age 18* b) *Median level of FeNO for each cluster from age 8 to 18* c) *Median level of FEV1/FVC z-score for each cluster from age 8 to age 18 at different time points* d) *Proportion of current wheeze for each cluster from age 8 to age 18*

Different colours are used for different clusters, the median level for each cluster is displayed . Light green = cluster 1, dark green = cluster 2, yellow = cluster 3, red = cluster 4, orange = cluster 5.

**Replication in the independent Isle of Wight cohort.**

Analogous symptoms could not be retrieved for both questionnaires at age 10 as well as at age 18. To increase the comparability between the two models, we decided to include only the overlapping objective measures.

**Figure S6. Bayesian profile regression results for IOW.**

The 5 clusters solution is described along the variables used in the model. The solid line represents the median population level for lung function measures. For the categorical variables, each dot represents the percentages of positive answer/test result. In the final clustering allocation, 4 clusters were retrieved formed by 260, 193, 44, and 19 observations of which 19 (7.31%), 24 (12.44%), 42 (95.45%), and 18 (94.74%) children diagnosed with asthma.


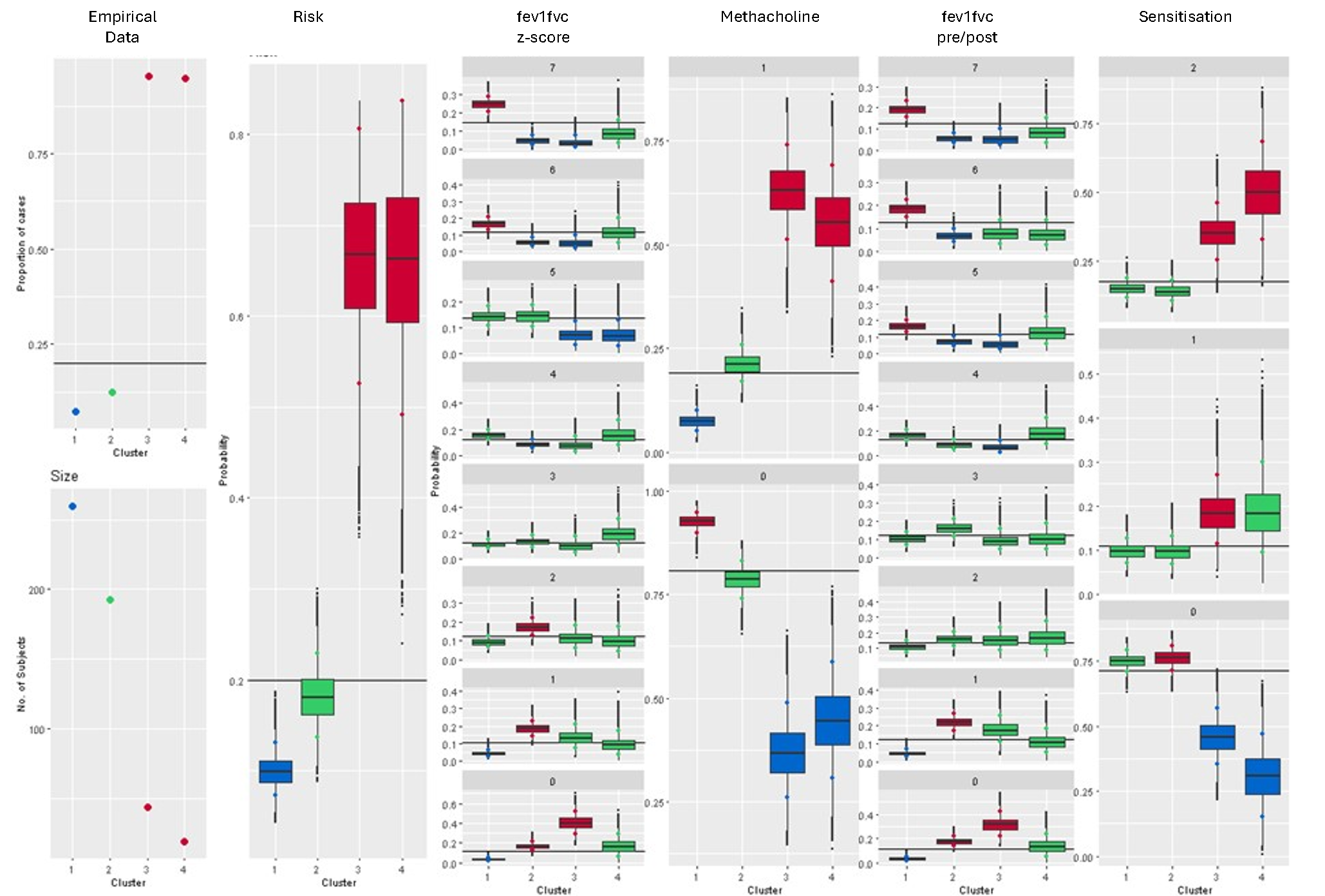


**Missing data handling**

We reasonably assume that coding misspecification occurred frequently for specific questions, and many missing values should have been marked as not applicable. Specifically, IOW questionnaires at age 18 were fulfilled via software in the clinic by the nurses. Although the system correctly labelled an answer as not applicable if displayed on the same view, it presumably did not block consequent views that may be not relevant for specific cases. Consequently, when a nurse answered in a negative way to specific questions, she/he could decide to skip non-meaningful questions that were then incorrectly labelled as missing instead of non-applicable. We decided to focus our attention on 4 specific questions that allowed us to reconstruct the outcome value of asthma 2 out of 3: Asthma Wheezing Episodes, Physician Diagnosed Asthma, Asthma Treatment, Wheezing 12 Months. Asthma Wheezing Episodes only had one Missing that was considered as missing. We then focused our attention on the remaining suspicious cases: Physician Diagnosed = Missing or don't know with Asthma Wheezing Episodes answers Asthma Treatment= Missing and Physician Diagnosed=1 Wheezing 12 Months= Missing or empty and Physician Diagnosed=1. Our actual analysis is focused on a small subset of observations and only a few subjects were considered in the subgroups specified. As we have used this information to derive the outcome as two out of three, for specific cases, considering the missing as non-applicable or as missing will not affect the outcome value. We removed the remaining unclear cases and proceed with the general assumption specified above. This led to the exclusion of 7 observations.

**Reference:**

1. Hastie, D.I., Liverani, S. & Richardson, S. Sampling from Dirichlet process mixture models with unknown concentration parameter: mixing issues in large data implementations. *Stat Comput* **25**, 1023-1037 (2015).

2. Liverani, S., Hastie, D.I., Azizi, L., Papathomas, M. & Richardson, S. PReMiuM: An R Package for Profile Regression Mixture Models Using Dirichlet Processes. *J Stat Softw* **64**, 1-30 (2015).

3. Liverani, S., Lavigne, A. & Blangiardo, M. Modelling collinear and spatially correlated data. *Spatial and spatio-temporal epidemiology* **18**, 63-73 (2016).

4. Papathomas, M., Molitor, J., Richardson, S., Riboli, E. & Vineis, P. Examining the joint effect of multiple risk factors using exposure risk profiles: lung cancer in nonsmokers. *Environ Health Perspect* **119**, 84-91 (2011).

5. Molitor, J., Papathomas, M., Jerrett, M. & Richardson, S. Bayesian profile regression with an application to the National survey of children's health. *Biostatistics* **11**, 484-498 (2010).

6. Molitor, J.*, et al.* Identifying vulnerable populations through an examination of the association between multipollutant profiles and poverty. *Environmental Science & Technology* **45**, 7754-7760 (2011).

7. Papaspiliopoulos, O. & Roberts, G.O. Retrospective Markov chain Monte Carlo methods for Dirichlet process hierarchical models. *Biometrika* **95**, 169-186 (2008).
